# Histone ubiquitination-related gene CUL4B promotes lung adenocarcinoma progression and cisplatin resistance

**DOI:** 10.3389/fgene.2023.1242137

**Published:** 2023-11-24

**Authors:** Yanjun Yin, Lifeng Zhang, Yinchuan Zeng, Diang Chen, Haien Guan, Guoping Ran, Kangming Du

**Affiliations:** ^1^ Department of Emergency, Hospital of Chengdu University of Traditional Chinese Medicine, Chengdu, China; ^2^ Hospital of Chengdu University of Traditional Chinese Medicine, Chengdu, China; ^3^ The General Hospital of Western Theater Command, Chengdu, China; ^4^ Center of Stem Cell and Regenerative Medicine, The People’s Hospital of Gaozhou, Gaozhou, China; ^5^ Chengdu Second People’s Hospital, Chengdu, China

**Keywords:** lung cancer, ubiquitination, histone, CUL4B, cisplatin resistance

## Abstract

**Background:** The role of the histone ubiquitination-related gene in the cisplatin resistance of lung adenocarcinoma (LUAD) remains an intricate subject.

**Methods:** We accessed transcriptome data of both wild type and cisplatin-resistant cells from the GSE108214 dataset, and garnered transcriptome and clinical data of LUAD patients from The Cancer Genome Atlas (TCGA) database. Utilizing the R software, we analyzed these public datasets in depth. Real-time Quantitative PCR (qPCR) was used to detect the RNA level of CUL4B. Effect of CUL4B on cell proliferation was evaluated using CCK8 and colony formation assay. Effect of CUL4B on cell invasion was evaluated using transwell assay. Cisplatin sensitivity was evaluated by calculating IC50.

**Results:** Our analysis shed light on the significance of the histone ubiquitination-related gene, CUL4B, in relation to cisplatin resistance and the overall survival rates of LUAD patients. Notably, CUL4B was found to be overexpressed in both lung cancer tissues and cells. Meanwhile, *in vitro* experiments indicated can CUL4B significantly promote the proliferation, invasion and migration of lung cancer cells. Furthermore, suppressing CUL4B expression led to a noticeable reduction in the IC50 value of cisplatin in lung cancer cells. A deep dive into biological enrichment analysis revealed that among patients exhibiting high CUL4B expression, there was a pronounced activation of the G2M checkpoint and the PI3K/AKT/mTOR signaling pathways. Immune microenvironment analysis has revealed that patients with elevated CUL4B expression may exhibit increased infiltration of M2 macrophages, coupled with a reduced infiltration of CD8^+^ T cells and activated NK cells. Notably, we observed higher CUL4B expression among those who responded positively to immunotherapy.

**Conclusion:** These findings underscore the significance of CUL4B in the resistance to cisplatin in lung cancer, highlighting its potential as a therapeutic target.

## Introduction

Lung cancer ranks among the most prevalent cancers worldwide, frequently leading to cancer-related fatalities (Collins et al., 2007; Nasim et al., 2019). Chemotherapy, particularly preoperative chemotherapy, remains a standard treatment approach for non-small cell lung cancer (NSCLC). Common drugs include cisplatin and taxanes, as well as Etoposide, Gemcitabine, vinorelbine and pemetrexed (Nagasaka and Gadgeel, 2018; Chaft et al., 2021). However, studies indicate that resistance rates for cisplatin and taxanes stand at approximately 63% and 43%, respectively (Rosell et al., 2002). While platinum-based chemotherapy has enhanced the long-term survival rates of NSCLC patients, its short-term efficacy is limited due to high toxicity, and it frequently results in drug resistance (Zhou et al., 2021). Consequently, cisplatin resistance has emerged as a principal factor in the mortality of NSCLC patients.

Given cisplatin’s pivotal role in lung cancer therapy, several researchers have dedicated their efforts to investigate its resistance and identify potential therapeutic targets (Kryczka et al., 2021). Zhang et al. identified that TRIM6 targets SLC1A5, influencing ferroptosis and chemosensitivity (Zhang et al., 2023). Similarly, Xia et al. highlighted that RECQL5 fosters NSCLC metastasis and contributes to cisplatin resistance (Xia et al., 2021). Ray et al. uncovered a connection between nicotine and the modulation of cisplatin resistance (Ray et al., 2022). Additionally, Hou et al. showed that FAM60A activates SKP2, thereby intensifying cisplatin resistance (Hou et al., 2020). He et al. determined the significance of FEN1 in both the progression of NSCLC and cisplatin resistance (He et al., 2017). Recently, with the advancements in bioinformatics, there’s been an upsurge in accessible data and refined algorithmic frameworks. This evolution equips researchers with the tools to efficiently pinpoint and validate potential biological targets (Taguchi, 2023).

Here, we shed light on the significance of the histone ubiquitination-related gene, CUL4B, in relation to cisplatin resistance and the overall survival rates of LUAD patients. Notably, CUL4B was found to be overexpressed in both lung cancer tissues and cells. Meanwhile, *in vitro* experiments indicated can CUL4B significantly promote the proliferation, invasion and migration of lung cancer cells. Furthermore, suppressing CUL4B expression led to a noticeable reduction in the IC50 value of cisplatin in lung cancer cells. A deep dive into biological enrichment analysis revealed that among patients exhibiting high CUL4B expression, there was a pronounced activation of the G2M checkpoint and the PI3K/AKT/mTOR signaling pathways. Immune microenvironment analysis has revealed that patients with elevated CUL4B expression may exhibit increased infiltration of M2 macrophages, coupled with a reduced infiltration of CD8^+^ T cells and activated NK cells. Notably, we observed higher CUL4B expression among those who responded positively to immunotherapy.

## Materials and methods

### Data acquisition and bioinformatics analysis

The open-access transcriptome data for cisplatin-resistant and wild-type A549 cells were sourced from the Gene Expression Omnibus (GEO) database under the GSE108214 project (Sarin et al., 2018). For this project, the expression matrix was obtained through the “Series Matrix File(s)” link and subsequently annotated using the GPL17077 platform. Additionally, we procured the transcriptome, clinical and genomic data for lung adenocarcinoma (LUAD) patients from The Cancer Genome Atlas Program (TCGA) database, specifically the STAR—Counts dataset. Prior to any analysis, we ensured all data underwent preprocessing. Limma package was utilized to conduct the differentially expressed genes (DEGs) analysis (threshold = |logFC| > 1 and adj P.value <0.05) (Ritchie et al., 2015). Kaplan-Meier survival curves were employed to analyze prognosis disparities between various groups (Rich et al., 2010). We derived the gene list associated with histone ubiquitination from the Gene set enrichment analysis (GSEA) project (Subramanian et al., 2005). Also, GSEA was undertaken to delineate the biological variances between patients exhibiting high or low CUL4B expression (the threshold of FDR = 0.25). More specifically, the Gene Set Variation Analysis (GSVA) was employed to ascertain the relative enrichment score of particular pathways (Hänzelmann et al., 2013). GSVA, a nuanced gene enrichment method, facilitates pathway-centric analyses of molecular data by transitioning the functional units from individual genes to gene sets. Our reference for this was the Hallmark gene set (Liberzon et al., 2015). For GSEA analysis, it can determine whether the functional annotation gene set has undergone significant changes in different samples. The CIBERSORT algorithm was used to quantify the immune microenvironment of LUAD tissue based on the transcriptional profile data (Chen et al., 2018). This deconvolution algorithm infers the cellular proportions in different samples based on bulk RNA data across varying data structures. Single-cell data for LUAD samples were sourced from the Tumor Immune Single-cell Hub (TISCH) project, including GSE131907 and GSE148071 projects (Sun et al., 2021). Lastly, the Tumor Immune Dysfunction and Exclusion (TIDE) algorithm was leveraged to gauge the immunotherapy response in LUAD patients (Fu et al., 2020). The baseline information of the enrolled patients was shown in Table 1.

**TABLE 1:** The baseline information of the TCGA patients enrolled in this study.

Clinical features		Number	Percentage (%)
Gender	Female	280	53.6
	Male	242	46.4
Age	< = 65	241	46.2
	>65	262	50.2
	Unknown	19	3.6
Stage	Stage I	279	53.4
	Stage II	124	23.8
	Stage III	85	16.1
	Stage IV	26	5.0
	Unknown	8	1.5
Tstage	T1	172	33.0
	T2	281	53.8
	T3	47	9.0
	T4	19	3.6
	Unknown	3	0.1
Mstage	M0	353	67.6
	M1	25	4.8
	Unknown	144	27.6
Nstage	N0	335	64.2
	N1	98	18.8
	N2-3	77	14.8
	Unknown	13	2.5

### Immunohistochemistry (IHC) and fluorescence

The IHC image and cell fluorescence were obtained from The Human Protein Atlas project (Uhlén et al., 2015). We used “CUL4B” as the specific search term, retrieving results from three search categories: “TISSUE,” “PATHOLOGY,” and “SUBCELL.”

### Cell lines and cell transfection

One normal cell line (BEAS-2B) and three lung cancer cell lines (A549, H1299 and H460) were purchased from the Cell Bank of Culture of the Chinese Academy of Sciences. The A549-Res (resistant to cisplatin) cell line was purchased from Shanghai MEIXUAN Biological Science and Technology Co, Ltd. All these cell lines were cultured under regular conditions: 5% CO_2_ and 37°C. The selection of culture medium is RPMI-1640. Cell transfection was conducted according to the standard process. The target sequence of shRNA-CUL4B were: sh-CUL4B#1, 5’-GGT​GCT​GCT​AAT​GTT​TAA​T-3’; sh-CUL4B#2: 5’-GGC​AGC​ACT​ATT​GTA​ATT​A-3’; sh-CUL4B#3: 5’-CCA​CCC​AGA​AGT​CAT​TAA​T-3’.

### Real-time quantitative PCR (qPCR)

The extraction of total RNA was performed using the total RNA extraction reagent according to the corresponding protocol and then reverse transcribed into cDNA. Detction of qPCR was conducted under the Sybr green system. The primer used were: CUL4B, forward, 5’-ACT​CCT​CCT​TTA​CAA​CCC​AGG-3’. Reverse, 5’-TCT​TCG​CAT​CAA​ACC​CTA​CAA​AC-3’; GAPDH, forward, 5’-GGA​GCG​AGA​TCC​CTC​CAA​AAT-3’, reverse, 5’-GGC​TGT​TGT​CAT​ACT​TCT​CAT​GG-3’.

### Detection of cell proliferation ability

CCK8 and colony formation were utilized to detect the cell proliferation ability, which were performed based on the standard process.

### Detection of cell invasion ability

Transwell assay was used to detect the cell invasion ability of cells, which was conducted according to the standard process (Chen et al., 2020).

### Detection of cisplatin IC50

According to the previous study, the IC50 of cisplatin was detected (Heinze et al., 2021).

### Western blot

Cells were lysed to extract proteins, whose concentrations were determined using the BCA assay. Proteins were then subjected to SDS-PAGE and transferred onto a nitrocellulose or PVDF membrane. After blocking non-specific binding sites with 5% milk or BSA, the membrane was incubated with the primary antibody (CUL4B, proteintech, 1:2000; GAPDH, proteintech, 1:50000) overnight and subsequently with an HRP-conjugated secondary antibody. Following washes, protein bands were visualized using ECL.

### Apoptosis detection

Cells were harvested, washed with PBS, and resuspended in binding buffer. They were then treated with Annexin V-FITC and propidium iodide (PI) following the manufacturer’s protocol. After incubation, the samples were analyzed by flow cytometry. Cells were gated to exclude debris, and based on staining, they were categorized as early apoptotic, late apoptotic/dead, or non-apoptotic. The percentage of cells in each apoptosis stage was determined using software.

### Tumor formation experiment in nude mice

The animal experiments conducted in this study have been approved by the Hospital Ethics Committee (2023-0211). Male nude mice (6–8 weeks old) were acclimated and kept under controlled conditions. Cells, suspended in a PBS-Matrigel mix, were subcutaneously implanted in the mice. Tumor growth was monitored and measured using calipers. Mice were euthanized when tumors reached a specified size, and the tumors were processed for histology. These tumors were then sectioned and subjected to Ki67 immunostaining to assess proliferation. The Ki67 proliferation index was quantified by counting positive cells in random microscopic fields and calculating their proportion to the total cells.

### Statistical analysis

All statistical analyses were conducted using the R programming language and GraphPad Prism 8 software. A statistical significance threshold was established at 0.05. Depending on the distribution of the data, various statistical methodologies were employed to ensure accurate and robust results: For normally distributed continuous data, we utilized the Student’s t-test; For continuous data that did not conform to a normal distribution, the Wilcoxon rank-sum test (also known as the Mann-Whitney U test) was used; For comparisons involving more than two groups with normally distributed data, we applied one-way or two-way ANOVA as appropriate, followed by *post hoc* tests; For categorical data, we employed the Chi-square test or Fisher’s exact test, depending on the sample size.

## Results

### Identification of histone ubiquitination-related genes associated with cisplatin resistance

**FIGURE 1:**
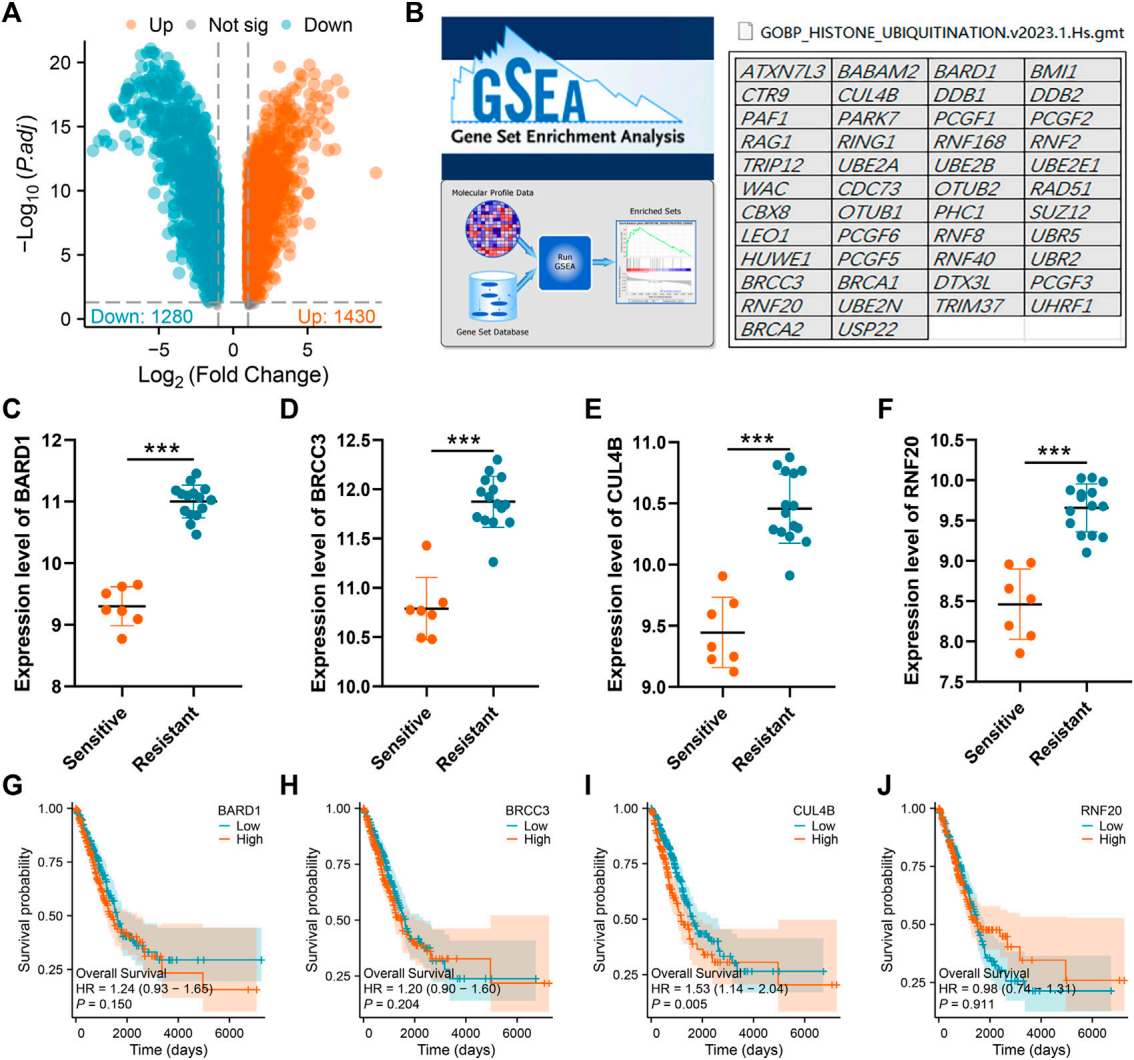
Identification of histone ubiquitination-related genes associated with cisplatin resistance. Notes: **(A)**: DEGs analysis was performed between wile type and cisplatin-resistant A549 cells; **(B)**: The gene list of histone ubiquitination-related genes was obtained from the GSEA project; **(C–F)**: The expression level of BARD1, BRCC3, CUL4B and RNF20 in cisplatin sensitive and resistant A549 cells, *** = *p* < 0.001; **(G)**: Kaplan-Meier survival curves in patients with high and low BARD1 expression; **(H)**: Kaplan-Meier survival curves in patients with high and low BRCC3 expression; **(I)**: Kaplan-Meier survival curves in patients with high and low CUL4B expression; **(J)**: Kaplan-Meier survival curves in patients with high and low RNF20 expression.

We initiated our study by collecting transcriptional data from the GSE108214 project. The sequential steps in the data preprocessing are delineated in Supplementary Figure S1. Specifically, Supplementary Figure S1A presents the original data distribution, while Supplementary Figure S1B depicts the distribution after preprocessing. From the preprocessed data, we discerned seven cisplatin-sensitive samples and 15 resistant ones. A subsequent analysis of DEGs between these samples revealed 1,280 genes that were downregulated and 1,430 that were upregulated in the cisplatin-resistant samples compared to the sensitive ones, as visualized in Figure 1A. Next, from the GSEA project, we compiled a list of genes implicated in the histone ubiquitination process. This list includes ATXN7L3, BABAM2, BARD1, BMI1, CTR9, CUL4B, DDB1, DDB2, PAF1, PARK7, PCGF1, PCGF2, RAG1, RING1, RNF168, RNF2, TRIP12, UBE2A, UBE2B, UBE2E1, WAC, CDC73, OTUB2, RAD51, CBX8, OTUB1, PHC1, SUZ12, LEO1, PCGF6, RNF8, UBR5, HUWE1, PCGF5, RNF40, UBR2, BRCC3, BRCA1, DTX3L, PCGF3, RNF20, UBE2N, TRIM37, UHRF1, BRCA2, and USP22 (Figure 1B). Upon further analysis, we found that four histone ubiquitination-related molecules were differentially expressed between samples sensitive and resistant to cisplatin, BRAD1, BRCC3, CUL4B and RNF20 (Figures 1C–F and Supplementary Figure S2). Remarkably, all four molecules, BRAD1, BRCC3, CUL4B, and RNF20, were upregulated in cisplatin-resistant samples (Figures 1C–F). Additionally, Kaplan-Meier survival curves highlighted that among these, only CUL4B had a significant correlation with the prognosis of LUAD patients (Figures 1G–J, BRAD1: *p* = 0.15; BRCC3: *p* = 0.204; CUL4B: *p* = 0.005; RNF20: *p* = 0.911).

### Expression pattern of CUL4B in LUAD

**FIGURE 2:**
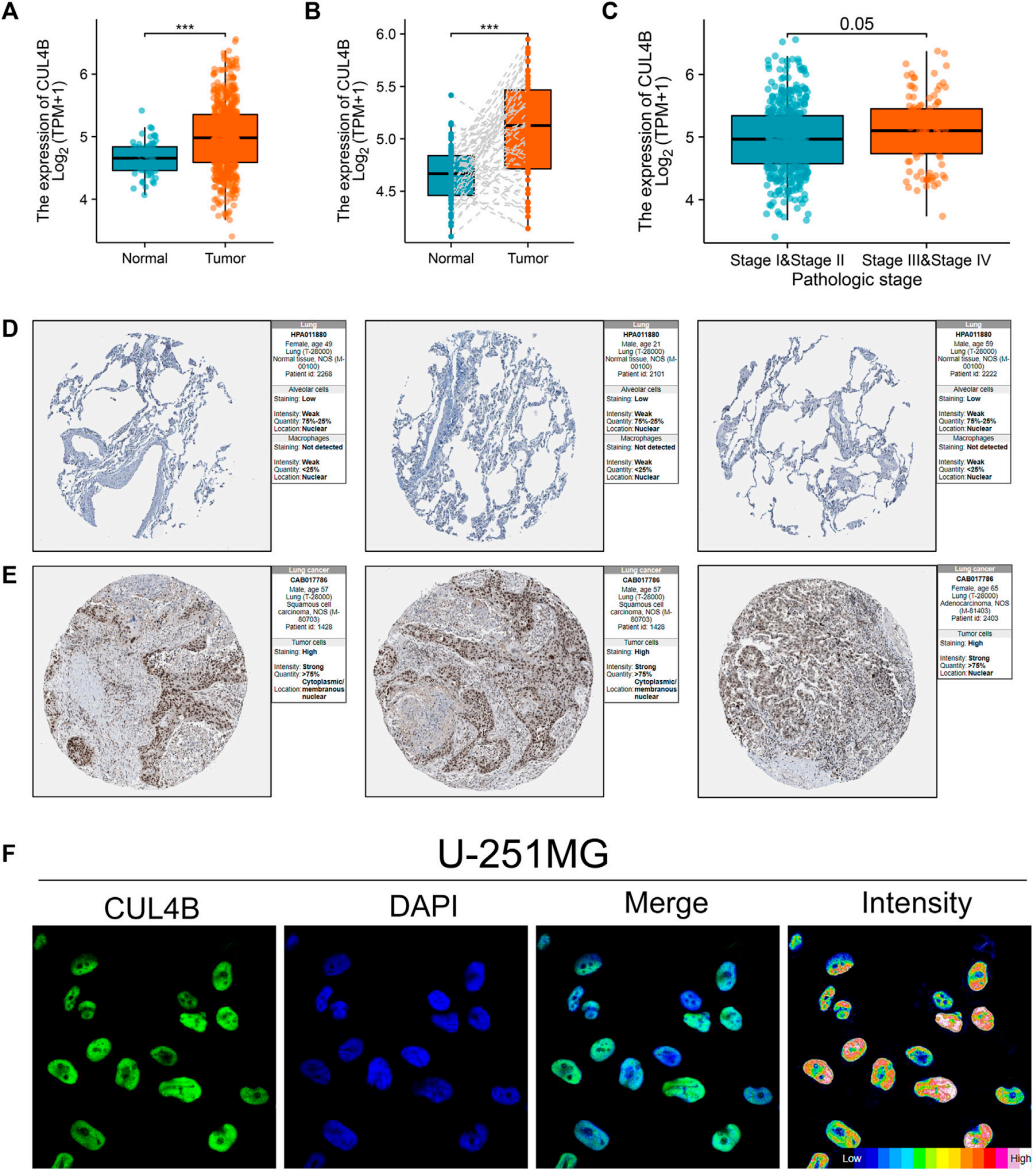
Expression pattern of CUL4B in LUAD. Notes: **(A)**: Expression pattern of CUL4B in LUAD and normal tissue, *** = *p* < 0.001; **(B)**: Expression pattern of CUL4B in paired LUAD and normal tissue; **(C)**: Expression pattern of CUL4B in Stage I/II and Stage III/IV; **(D)**: The IHC image of CUL4B of normal lung tissue; **(E)**: The IHC image of CUL4B of lung cancer tissue; **(F)**: Subcellular localization of CUL4B obtained from HPA database.

The aforementioned findings suggest that the histone ubiquitination-related molecule, CUL4B, plays a role in cisplatin resistance and impacts the prognosis of LUAD patients. Consequently, we chose CUL4B as our focal molecule for in-depth analysis. Consequently, we chose CUL4B as our focal molecule for in-depth analysis. We observed an upregulation of CUL4B in LUAD tissues (Figure 2A). This trend was also consistent in paired tissue analyses (Figure 2B). Clinical correlation studies highlighted that elevated CUL4B levels correlate with a more advanced clinical stage (Figure 2C; Stage III/IV vs. Stage I/II). Subsequently, we retrieved the IHC images of CUL4B from the HPA database, which further indicated heightened protein levels of CUL4B in lung cancer tissues (Figures 2D,E). Subcellular localization results revealed that CUL4B predominantly resides in the nucleoplasm (Figure 2F).

### CUL4B affects lung cancer cells proliferation, invasion and apoptosis

**FIGURE 3:**
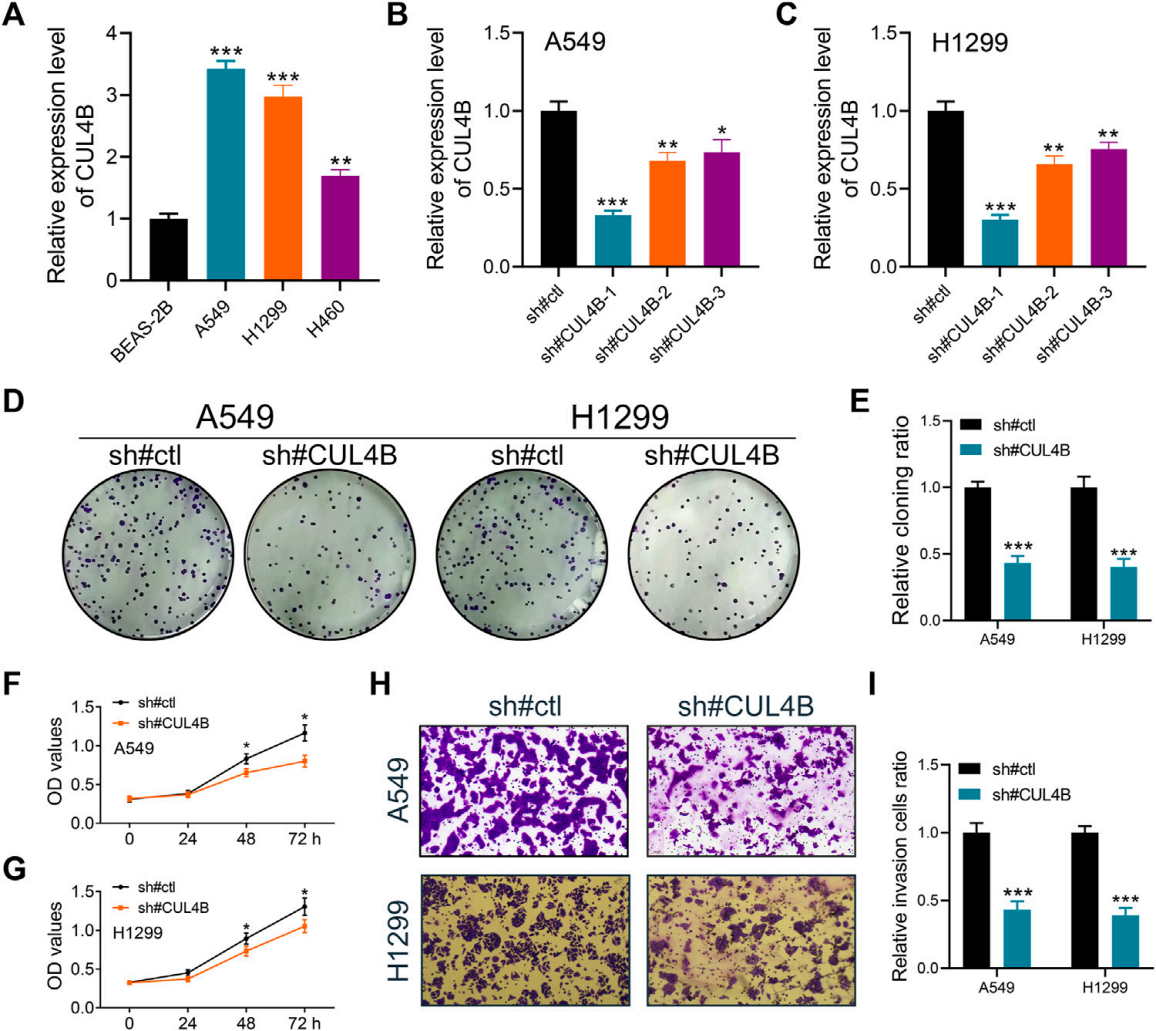
CUL4B promotes the proliferation, invasion and migration ability of lung cancer cells. Notes: **(A)**: Expression level of CUL4B in lung cancer cells, ** = *p* < 0.01, *** = *p* < 0.001; **(B, C)**: The knockdown efficiency of CUL4B was validated by qPCR, * = *p* < 0.05, ** = *p* < 0.01, *** = *p* < 0.001; **(D, E)** Colony formation assay was performed in control and CUL4B knockdown cells, *** = *p* < 0.001; **(F, G)**: CCK8 assay was performed in control and CUL4B knockdown cells, * = *p* < 0.05; **(H, I)**: Transwell assay was performed in control and CUL4B knockdown cells, *** = *p* < 0.001.

**FIGURE 4:**
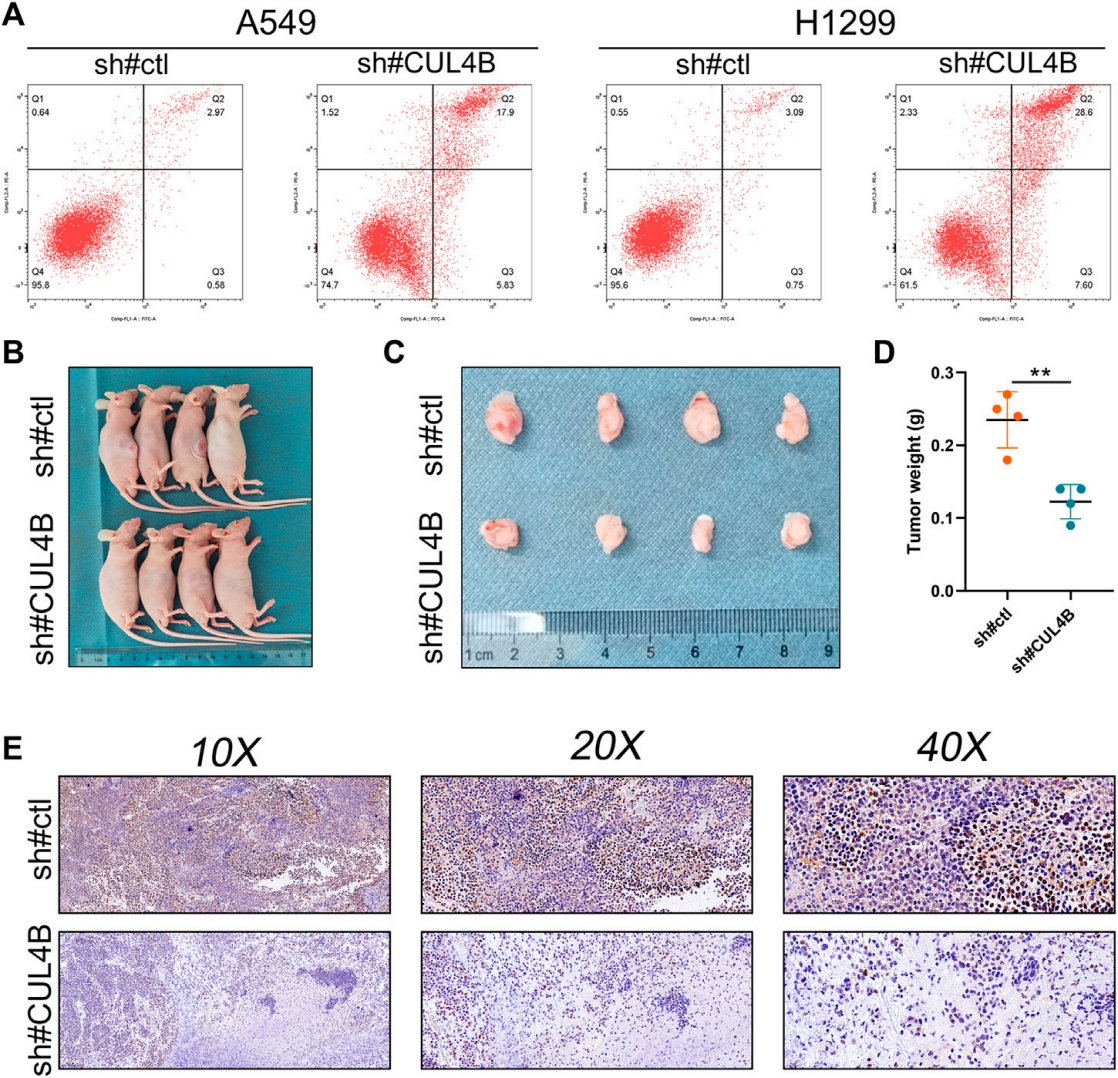
CUL4B inhibits cell apoptosis and promote tumor growth. Notes: **(A)**: Flow cytometry apoptosis detection in control and CUL4B knockdown cells; **(B, C)**: Tumor formation in nude mice was performed in control and CUL4B knockdown groups; **(D)**: The tumor weight of tumor body; **(E)**: Ki67 staining of the tumor body in control and CUL4B knockdown groups.

Additionally, we sought to elucidate the biological significance of CUL4B at the cellular level using various cell lines. Western blot and qPCR results indicated an upregulation of CUL4B in lung cancer cell lines (Figure 3A and Supplementary Figure S3). Subsequently, the efficacy of CUL4B knockdown was assessed using qPCR, revealing that sh-CUL4B#1 exhibited the most optimal knockdown efficiency (Figures 3B,C). Based on this, we proceeded with sh-CUL4B#2 for subsequent experiments. The colony formation assay demonstrated that suppressing CUL4B in A549 and H1299 cell lines markedly diminished the number of colonies (Figures 3D,E, *p* < 0.001). This trend was further corroborated by CCK8 assay results (Figures 3F,G). Transwell assay revealed that the knockdown of CUL4B can remarkably inhibit the cell invasion ability of A549 and H1299 cells (Figures 3H,I). Moreover, we explored the underlying effect of CUL4B on lung cancer cell apoptosis. Results showed that the inhibition of CUL4B can significantly promote the apoptosis of lung cancer cells (Figure 4A). Subsequently, we conducted xenograft experiments in nude mice to investigate the effects of CUL4B on the proliferation of lung cancer cells *in vivo*. The results revealed that tumors originating from the injection of CUL4B knockdown lung cancer cells had significantly lower weights compared to those from the control cells (Figures 4B–D). Additionally, Ki67 staining results demonstrated that tumors from CUL4B knockdown lung cancer cells exhibited a lower proportion of Ki67-positive cells compared to the control group (Figure 4E).

### CUL4B is associated with cisplatin resistance

**FIGURE 5:**
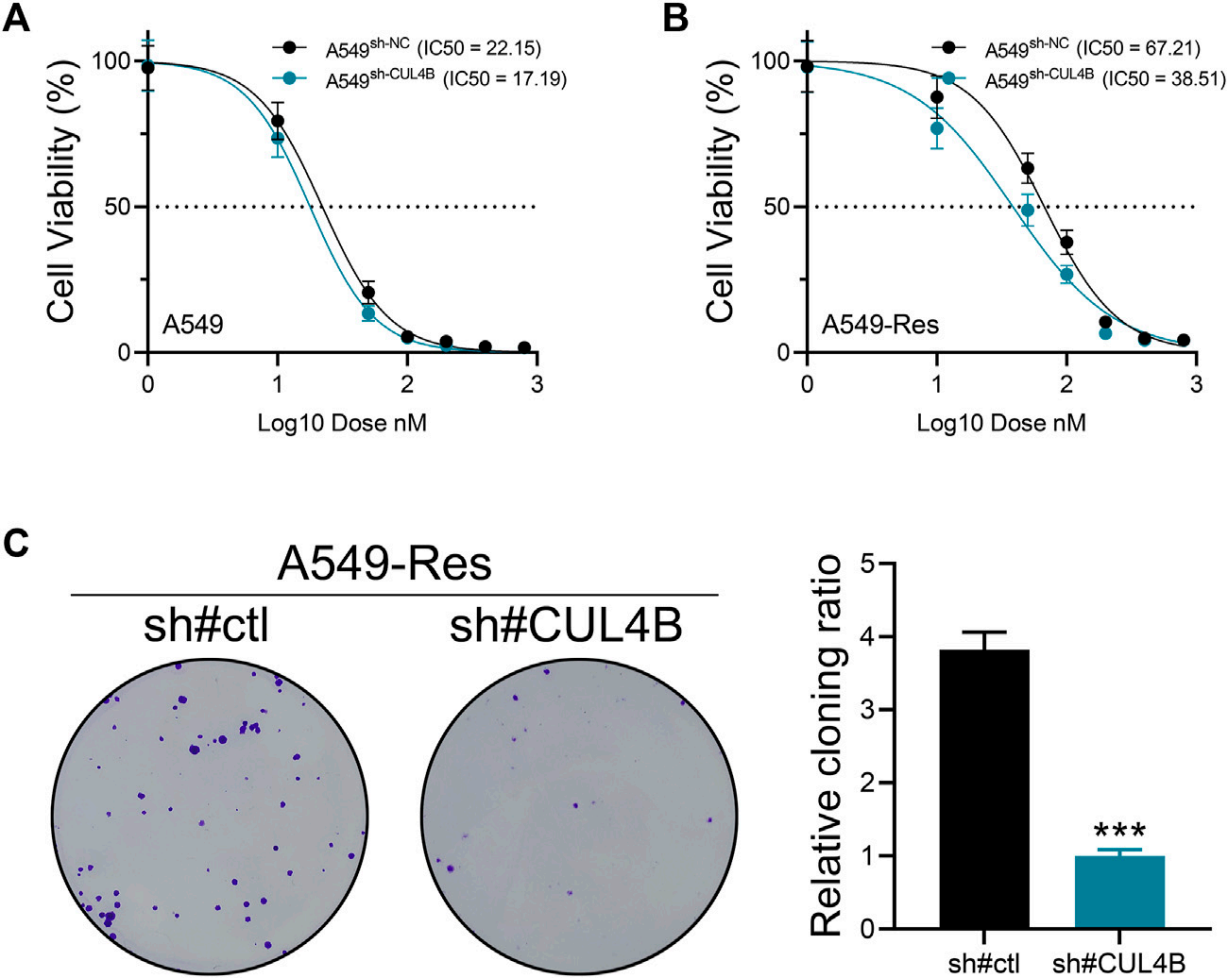
CUL4B affects cisplatin resistance of lung cancer cells. Notes: **(A)** The IC50 of cisplatin of control and CUL4B knockdown A549 cells; **(B)** The IC50 of cisplatin of control and CUL4B knockdown A549-Res cells; **(C)** Colony formation assay of control and CUL4B knockdown A549-Res cells, *** = *p* < 0.001.

We next aimed to determine the influence of CUL4B on cisplatin sensitivity. To this end, we measured the IC50 of cisplatin in both control and CUL4B-knockdown A549 cells (Figure 5A, wild type; Figure 5B, cisplatin-resistant). The data revealed that silencing CUL4B could lower the IC50 of cisplatin in A549 cells (A549-sh-NC: IC50 = 22.15, A549-sh-CUL4B: IC50 = 17.19; A549-Res-sh-NC: IC50 = 67.21, A549-Res-sh-CUL4B: IC50 = 38.51). Furthermore, we conducted a colony formation assay on cisplatin-resistant A549 cells treated with cisplatin. The findings suggest that inhibiting CUL4B markedly curtails the proliferation capability of cisplatin-resistant A549 cells (Figure 5C).

### Underlying biological enrichment of CUL4B

**FIGURE 6:**
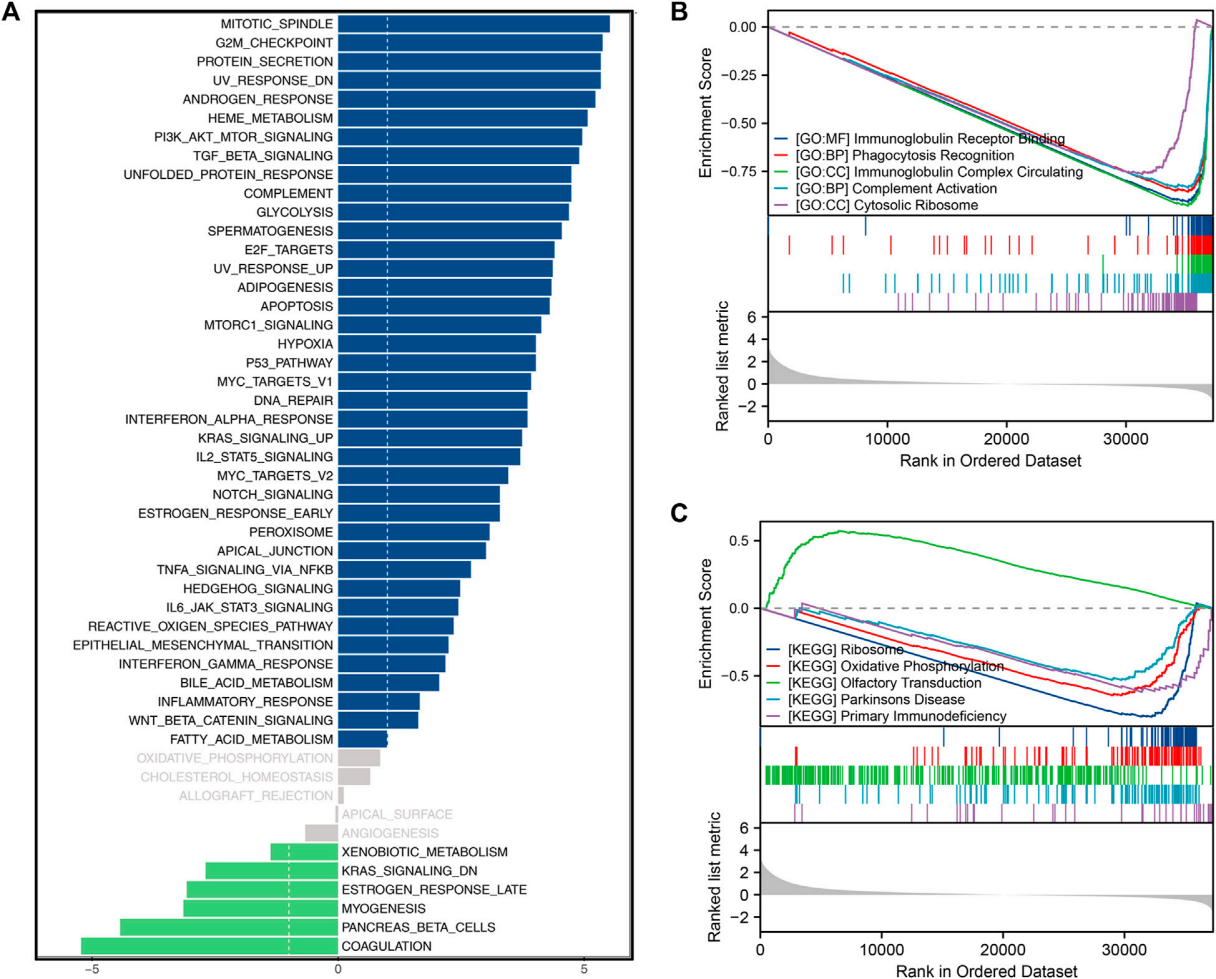
Biological enrichment analysis. Notes: **(A)**: GSVA analysis based on Hallmark gene set in patients with high and low CUL4B expression; **(B)**: GSEA analysis based on GO gene set in patients with high and low CUL4B expression; **(C)**: GSEA analysis based on KEGG gene set in patients with high and low CUL4B expression.

GSVA results revealed that in patients with elevated CUL4B expression, there was significant activation in pathways associated with the mitotic spindle, G2M checkpoint, protein secretion, UV response DN, androgen response, TGF-β signaling, PI3K/AKT/mTOR signaling, unfolded protein response, complement, and glycolysis (Figure 6A). GSEA analysis, grounded in GO terms, suggested associations between CUL4B and various functions including immunoglobulin receptor binding, phagocytosis recognition, circulation of immunoglobulin complexes, complement activation, and cytosolic ribosome (Figure 6B). Further GSEA insights, based on KEGG terms, indicated CUL4B’s involvement in processes related to the ribosome, oxidative phosphorylation, olfactory transduction, Parkinson’s disease, and primary immunodeficiency (Figure 6C). Meanwhile, we explored the correlation between CUL4B and genomic features. However, we found that CUL4B was not significantly correlated with TMB score, as well as MSI score (Supplementary Figure S4).

### Immune microenvironment and immunotherapy analysis

**FIGURE 7:**
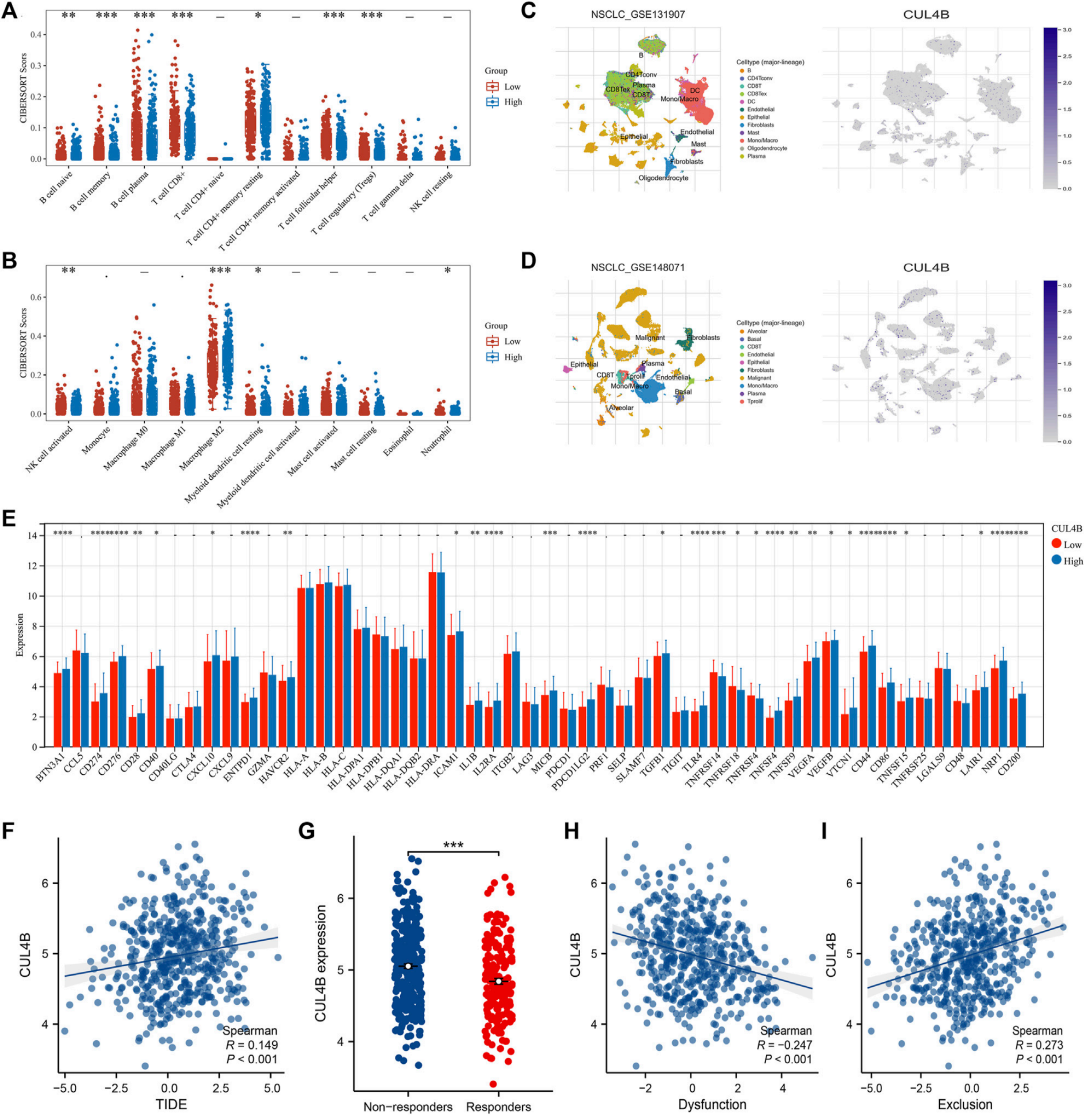
Effect of CUL4B on immune microenvironment. Notes: **(A, B):** CIBEROSRT algorithm was performed in patients with high and low CUL4B, * = *p* < 0.05, ** = *p* < 0.01, *** = *p* < 0.001; **(C, D)**: Single-cell analysis of CUL4B in lung cancer tissue; **(E)**: Expression pattern of multiple immune checkpoint genes in patients with high and low CUL4B expression, * = *p* < 0.05, ** = *p* < 0.01, *** = *p* < 0.001, **** = *p* < 0.0001; **(F)**: Correlation between TIDE score and CUL4B; **(G)**: Expression level of CUL4B in immunotherapy responders and non-responders, *** = *p* < 0.001; **(H)**: Correlation between CUL4B and immune dysfunction; **(I)**: Correlation between CUL4B and immune exclusion.

We utilized the CIBERSORT algorithm to quantify the immune microenvironment within LUAD tissues (Figures 7A,B). The data suggested that patients with elevated CUL4B expression likely exhibit increased infiltration of M2 macrophages, juxtaposed with decreased infiltration levels of CD8^+^ T cells and activated NK cells (Figures 7A,B). Single-cell analysis, drawing from datasets GSE131907 and GSE148017, revealed a widespread distribution of CUL4B across the majority of cells (Figures 7C,D). We investigated the relationship between CUL4B and various immune checkpoint genes. Results showed that the BTN3A1, CD274, CD276, CD28, CD40, CXCL10, ENTPD1, HAVCR2, ICAM1, IL1B, IL2RA, MICB, PDCD1LG2, TGFB1, TLR4, TNFSF4, TNFSF9, VEGFA, VEGFB, VTCN1, CD44, CD86, TNFSF15, LAIR1, NRP1, and CD200 were upregulated, yet TNFRSF14, TNFRSF18 and TNFRSF4 were downregulated in patients with high CUL4B expression (Figure 7E). TIDE analysis indicates a positive correlation between TIDE and CUL4B expression (Figure 7F, R = 0.149, *p* < 0.001). Moreover, we noticed a higher expression level of CUL4B in the immunotherapy responders (Figure 7G). Interestingly, we noticed that CUL4B is negatively correlated with immune dysfunction, but positively correlated with immune exclusion (Figure 7H: immune dysfunction, cor = −0.247, *p* < 0.001; Figure 7I: immune exclusion, cor = 0.273, *p* < 0.001). The whole flow chart of the study was shown in Supplementary Figure S5.

## Discussion

Globally, lung cancer ranks as one of the most lethal malignancies (Beattie, 1974; Stahel, 1994). Over recent years, both the incidence and mortality rates of lung cancer have been on the rise, particularly among males (Rivera and Stover, 2004). Intriguingly, even within the same country, different regions often report varying incidence rates. The onset of lung cancer results from the interplay of numerous factors, making its etiology intricate. Factors such as nicotine exposure, alcohol consumption, genetics, obesity, among others, play a role in its development (de Groot and Munden, 2012; Qi et al., 2021). Despite significant advancements in medical technology, lung cancer treatment outcomes frequently fall short of expectations. For stages I and II NSCLC patients, simple surgical resection or surgical-based comprehensive treatment are the first and best treatment option (Howington et al., 2013). However, for those in the advanced stages, surgical interventions often prove less beneficial. Platinum-containing chemotherapy has always been the preferred option for lung cancer chemotherapy (Gadgeel et al., 2020). Cisplatin is the first platinum drug approved for cancer treatment, which can be used to treat various malignant tumors and cancers. However, during the treatment of lung cancer, the occurrence of cisplatin resistance in patients has become the biggest bottleneck (Konoshenko et al., 2022). The resistance mechanism of lung cancer to cisplatin is as multifaceted and intricate as the disease’s onset. Hence, the pressing need arises to identify novel and effective targets that can influence cisplatin resistance.

Here, we shed light on the significance of the histone ubiquitination-related gene, CUL4B, in relation to cisplatin resistance and the overall survival rates of LUAD patients. Notably, CUL4B was found to be overexpressed in both lung cancer tissues and cells. Meanwhile, *in vitro* experiments indicated can CUL4B significantly promote the proliferation, invasion and migration of lung cancer cells. Furthermore, suppressing CUL4B expression led to a noticeable reduction in the IC50 value of cisplatin in lung cancer cells. A deep dive into biological enrichment analysis revealed that among patients exhibiting high CUL4B expression, there was a pronounced activation of the G2M checkpoint and the PI3K/AKT/mTOR signaling pathways. Immune microenvironment analysis has revealed that patients with elevated CUL4B expression may exhibit increased infiltration of M2 macrophages, coupled with a reduced infiltration of CD8^+^ T cells and activated NK cells. Notably, we observed higher CUL4B expression among those who responded positively to immunotherapy.

The covalent modification of histones is instrumental in regulating gene transcription and fundamentally shapes the life processes of organisms. Current research indicates that ubiquitination modifications of histones are prevalent across cellular structures (Figlia et al., 2020). The effects of histone ubiquitination, whether activating or inhibiting gene transcription, can vary based on the specific cell type or its life stage. This process has profound implications for biological growth, developmental trajectories, and adaptative responses to stimuli (Mattiroli and Penengo, 2021). The intrigue around the implications of histone ubiquitination modifications in cancer has been steadily growing. For example, Deng et al. revealed that HDAC6 has the ability to mediate the ubiquitination of AKAP12, a mechanism that augments the metastatic capabilities of colon cancer (Deng et al., 2022). Similarly, Li et al. deduced that the influential histone methyltransferase, EZH2, undergoes modulation by PRMT1, a shift that amplifies breast cancer metastasis (Li et al., 2020). Our study identified that the histone ubiquitination-related gene CUL4B is involved in cisplatin resistance and might be an underlying target for LUAD patients. Some studies also reported the role of CUL4B in cancers. For example, Wang et al. found that CUL4B could lead to tamoxifen resistance to breast cancer by regulating the miR-32-5p/ER-α36 axis (Wang et al., 2021). Qi et al. indicated that CUL4B could facilitate gastric cancer progression by targeting HER2 (Qi et al., 2018).

In patients exhibiting high CUL4B expression, we observed a pronounced upregulation of certain carcinogenic pathways, notably the G2M checkpoints and the PI3K/AKT/mTOR signaling pathways. DNA damage response serves as a critical cellular mechanism to uphold genomic stability against the persistent onslaught of both endogenous and exogenous DNA damage (Spitz et al., 2003). One of the important mechanisms of lung cancer, as a disease characterized by sustained excessive cell division, is the abnormality of the cell cycle process. In the cell cycle, tumor cells predominantly rely on the G2/M checkpoint, a pivotal cell cycle juncture, to avert potential mitotic catastrophes (Lai et al., 2022). Almasi et al. demonstrated that TRPM2 knockdown can trigger the JNK pathways, consequently inducing G2/M phase arrest (Almasi et al., 2019). The PI3K/AKT signaling pathway, ubiquitously present in human cells, plays a central role in modulating regular cellular physiological activities. In a variety of malignant tumor cells, PI3K/AKT signaling pathway is often in an overactive state (Fresno Vara et al., 2004). Specifically, regarding lung cancer, Yu et al. highlighted that baicalein could potentiate the sensitivity of A549 cells, mediated through the PI3K/AKT signaling axis (Yu et al., 2017).

Immune microenvironment analysis revealed that patients with elevated CUL4B expression tend to exhibit increased infiltration of M2 macrophages, contrasted with decreased infiltration of CD8^+^ T cells and activated NK cells. This influence of CUL4B on the lung cancer microenvironment might shed light on its association with adverse prognostic outcomes. M2 macrophages are frequently implicated in promoting tumor growth in solid malignancies. In lung cancer, Huang et al. found that lung cancer cells resistant to cisplatin can enhance M2 macrophage polarization through the Src/CD155/MIF axis (Huang et al., 2019). Further, Wei et al. observed that exosomes emanating from M2 macrophages could drive LUAD progression through the secretion of miR-942 (Wei et al., 2022). The continuous emergence of tumor immunotherapy methods based on CD8+T cells marks the progress of CD8+T cells in anti-tumor therapy, and their value and potential in clinical transformation are highly concerning (Dolina et al., 2021). Our results indicated that CUL4B might influence the immune microenvironment of LUAD tissue, further affecting cancer progression.

Despite our study being grounded in reliable analyses and subjected to meticulous experimental validation, there are still certain limitations that warrant attention. First and foremost, given the intricate nature of tissues and the overarching complexity of living organisms, analyses at the cellular level may not always accurately reflect the genuine scenario within the entire organism. Additionally, constrained by the limitations of algorithms and data quality, bioinformatics analysis results often cannot wholly align with the real biological processes. While we have embarked on a preliminary exploration of CUL4B’s influence on lung cancer and cisplatin, a deeper understanding of the underlying mechanisms remains to be further investigated.

## Data Availability

The raw data supporting the conclusion of this article will be made available by the authors, without undue reservation.

## Supplementary material

SUPPLEMENTARY FIGURE S1 Data preprocessing process Notes: **(A)**: Original data distribution; **(B)**: Processed data distribution.

SUPPLEMENTARY FIGURE S2 Intersection of histone ubiquitination-related molecules and DEGs between samples sensitive and resistant to cisplatin.

SUPPLEMENTARY FIGURE S3 Western blot was performed to detect the protein level of CUL4B in different cells.

SUPPLEMENTARY FIGURE S4 The correlation between CUL4B and genomic features Notes: **(A)**: The correlation between CUL4B and TMB score; **(B)**: The correlation between CUL4B and MSI score.

SUPPLEMENTARY FIGURE S5 The flow chart of the whole study.
