# Supplementary figures and images for: Histone ubiquitination-related gene CUL4B promotes lung adenocarcinoma progression and cisplatin resistance
Source: Front Genet. 2023 Nov 24;14:1242137. doi: 10.3389/fgene.2023.1242137 (PMC10704176; doi:10.3389/fgene.2023.1242137)

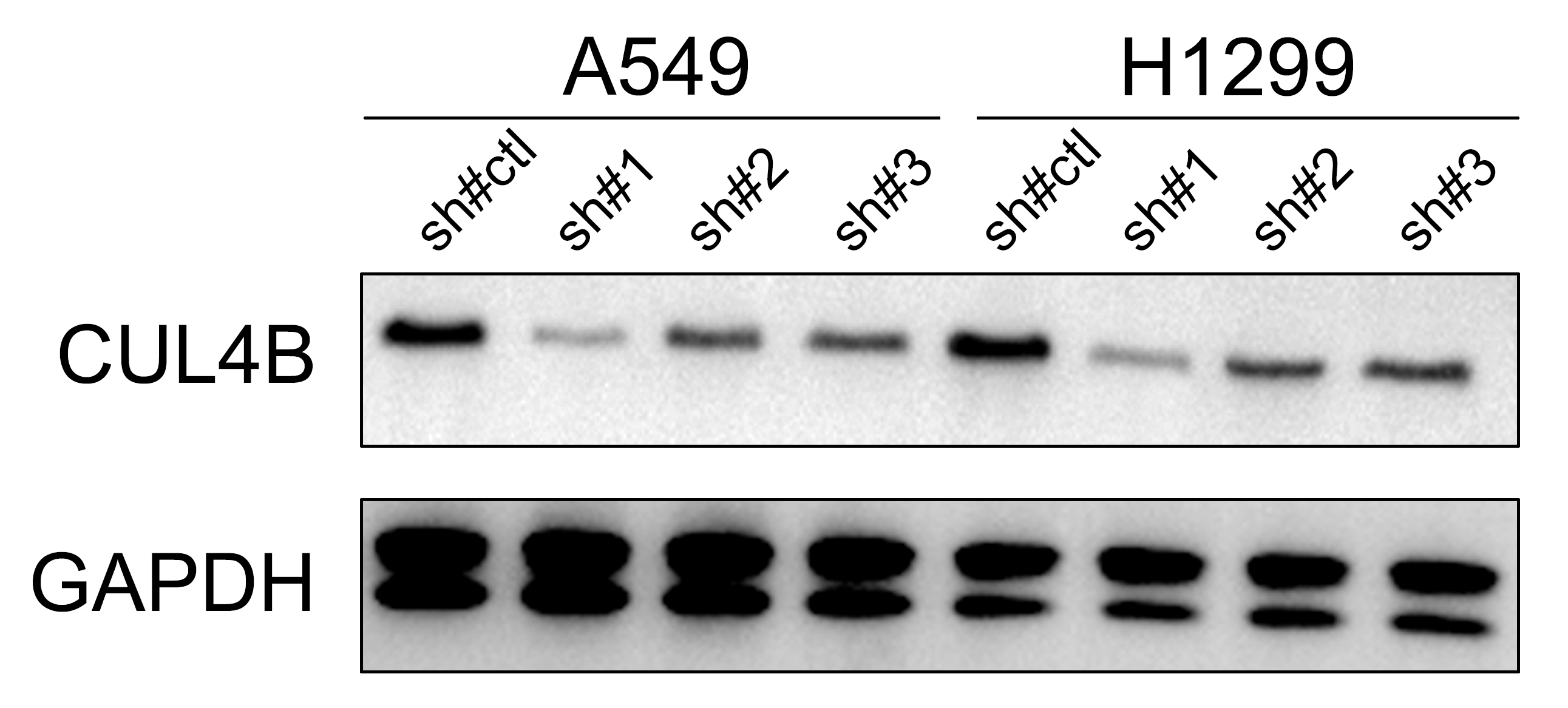

Supplement: Supplementary file 1 [file Image3.TIF]

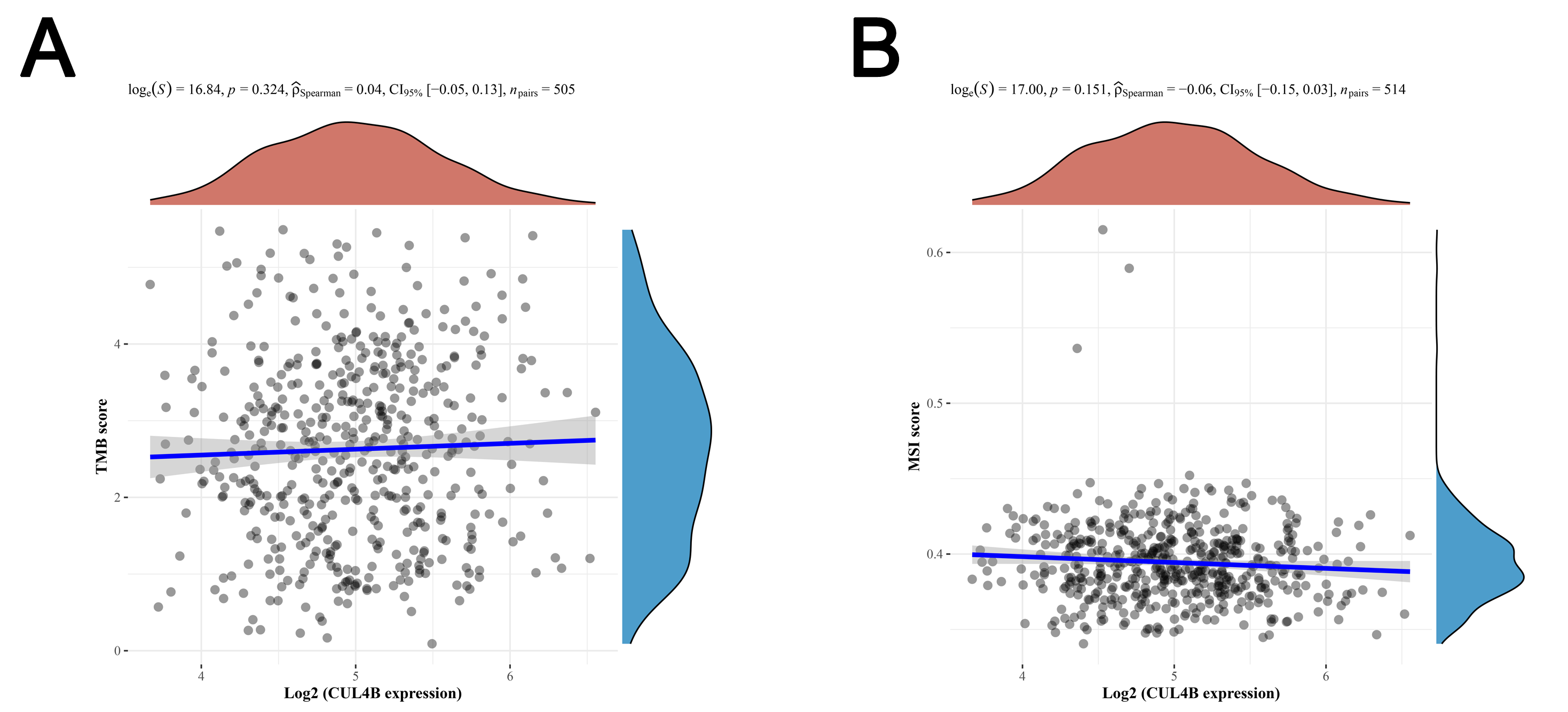

Supplement: Supplementary file 2 [file Image4.TIF]

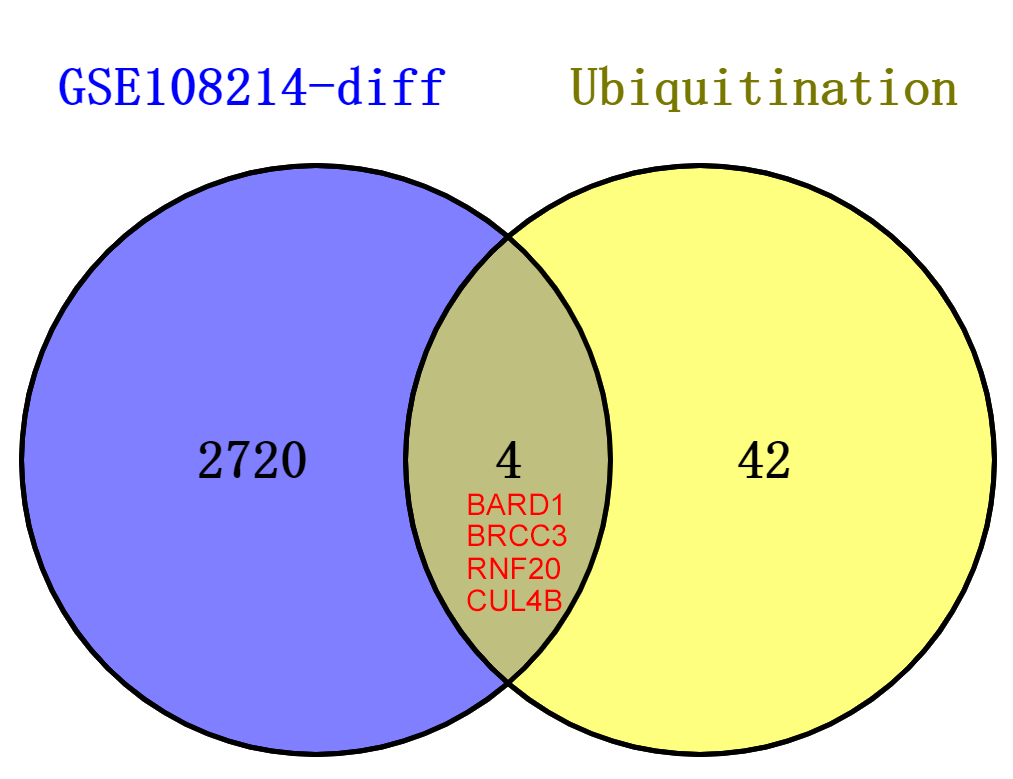

Supplement: Supplementary file 3 [file Image2.TIF]

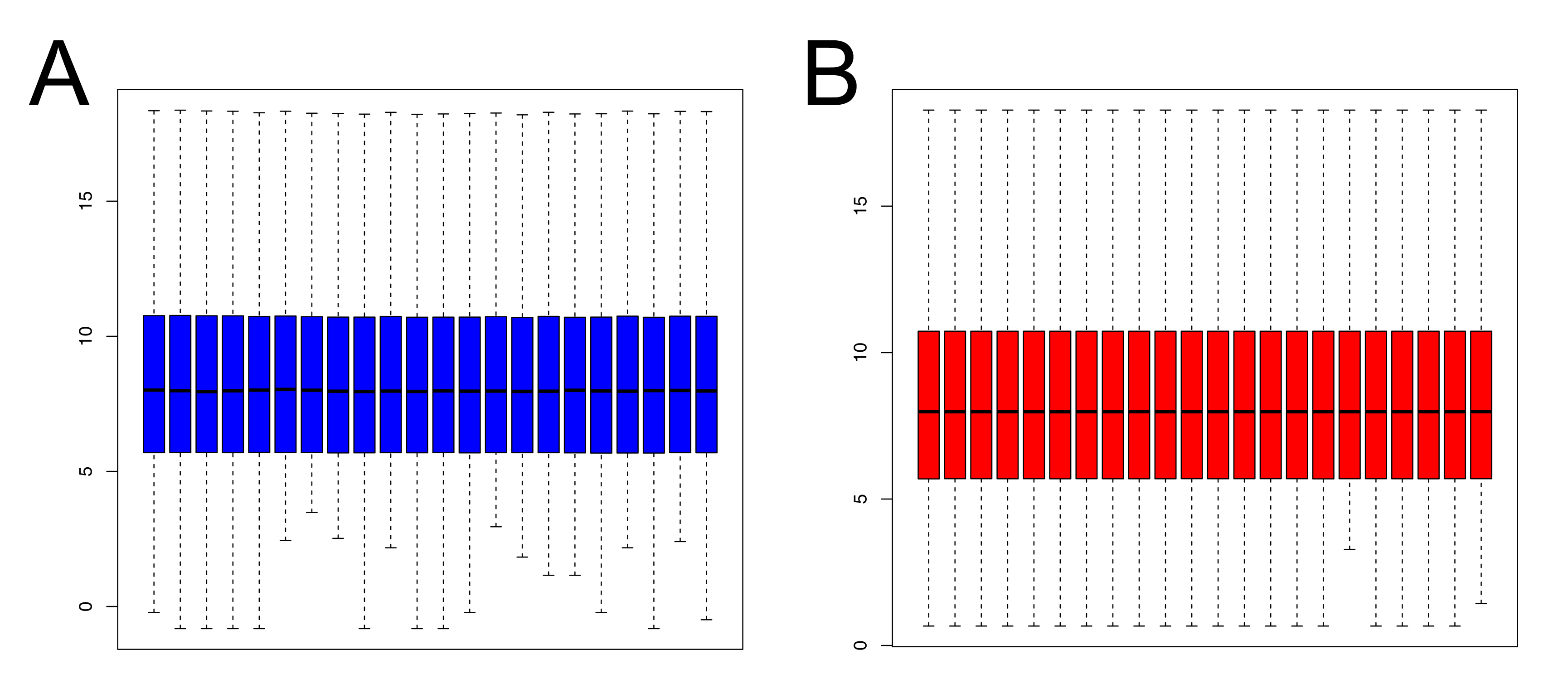

Supplement: Supplementary file 4 [file Image1.TIF]

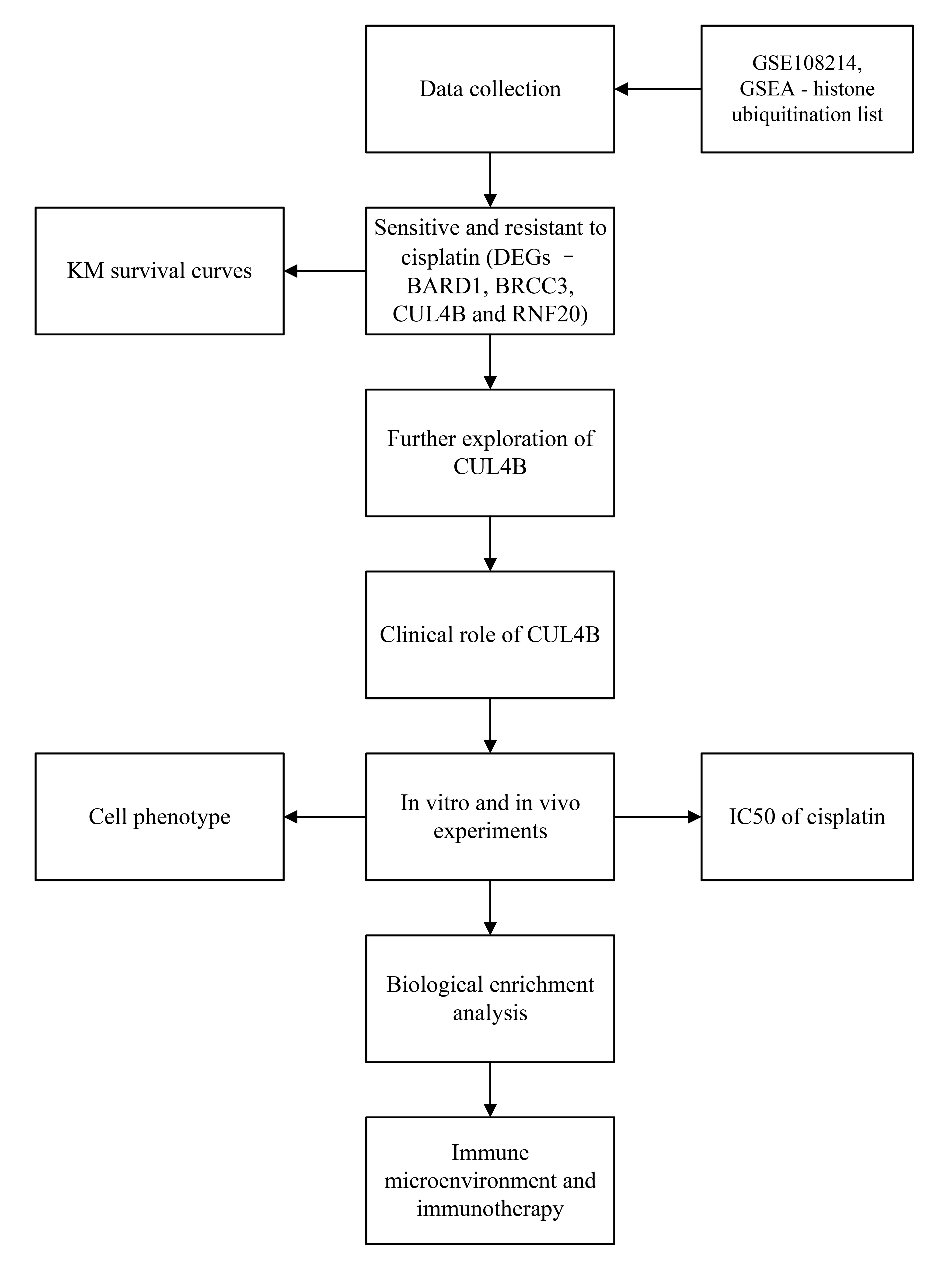

Supplement: Supplementary file 5 [file Image5.TIF]
